# Recent increase of surface particulate matter concentrations in the Seoul Metropolitan Area, Korea

**DOI:** 10.1038/s41598-017-05092-8

**Published:** 2017-07-05

**Authors:** Hyun Cheol Kim, Soontae Kim, Byeong-Uk Kim, Chun-Sil Jin, Songyou Hong, Rokjin Park, Seok-Woo Son, Changhan Bae, MinAh Bae, Chang-Keun Song, Ariel Stein

**Affiliations:** 1 Air Resources Laboratory, National Oceanic and Atmospheric Administration, College Park, MD USA; 2 Cooperative Institute for Climate and Satellites, University of Maryland, College Park, MD USA; 3 Department of Environmental and Safety Engineering, Ajou University, Suwon, South Korea; 4 Georgia Environmental Protection Division, Atlanta, GA USA; 5 National Centers for Environmental Prediction, National Oceanic and Atmospheric Administration, College Park, MD USA; 6 Korea Institute of Atmospheric Prediction System, Seoul, South Korea; 7 School of Earth and Environmental Sciences, Seoul National University, Seoul, South Korea; 8 National Institute of Environmental Research, Incheon, South Korea; 9 Korea Institute of Nuclear Safety, Daejeon, South Korea; 10 School of Urban and Environmental Engneering, Ulsan National Institute of Science and Technology, Ulsan, South Korea

## Abstract

Recent changes of surface particulate matter (PM) concentration in the Seoul Metropolitan Area (SMA), South Korea, are puzzling. The long-term trend of surface PM concentration in the SMA declined in the 2000s, but since 2012 its concentrations have tended to incline, which is coincident with frequent severe hazes in South Korea. This increase puts the Korean government’s emission reduction efforts in jeopardy. This study reports that interannual variation of surface PM concentration in South Korea is closely linked with the interannual variations of wind speed. A 12-year (2004–2015) regional air quality simulation was conducted over East Asia (27-km) and over South Korea (9-km) to assess the impact of meteorology under constant anthropogenic emissions. Simulated PM concentrations show a strong negative correlation (i.e. R = −0.86) with regional wind speed, implying that reduced regional ventilation is likely associated with more stagnant conditions that cause severe pollutant episodes in South Korea. We conclude that the current PM concentration trend in South Korea is a combination of long-term decline by emission control efforts and short-term fluctuation of regional wind speed interannual variability. When the meteorology-driven variations are removed, PM concentrations in South Korea have declined continuously even after 2012.

## Introduction

Regional air quality in the Seoul Metropolitan Area (SMA), South Korea, is a serious public concern, especially due to frequent haze events in recent years^1–3^. Understanding the cause and effect of air pollution in South Korea is complicated because air quality in East Asia is affected by numerous factors, including local and regional emissions, and meteorological and chemical interactions^4^. The SMA, which consists of Seoul, Incheon, and Gyeong-Gi, is a highly urbanized area with lots of emission sources in and around the area, such as traffic, industrial factories, and power generating facilities^5^. Often, this region’s air quality is affected not only by local emission sources, but also by the long-range transport of anthropogenic emissions from neighboring countries including China, and natural sources such as Asian dusts^6–8^ and wildfires from Mongolia and Russia^9, 10^.

**Figure 1:**
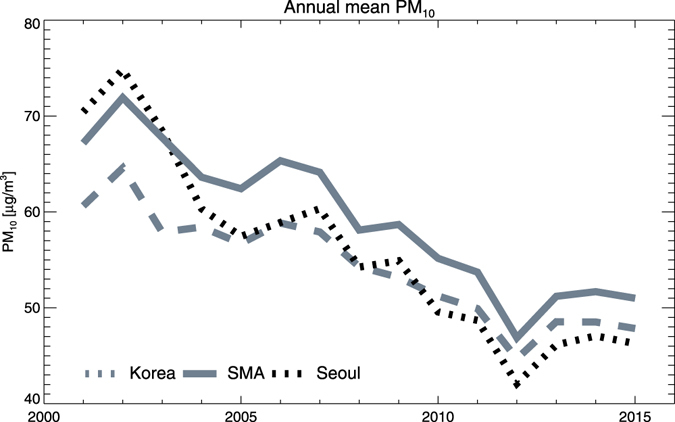
Time series of observed annual mean surface PM_10_ concentrations over Seoul (30 sites), the SMA (112 sites), and South Korea (247 sites) from 2001 to 2015. Box plots indicate 25^th^, 50^th^, and 75^th^ percentiles of the SMA PM concentrations.

Since the early 2000s, the South Korean government has legislated a special act to improve air quality in the SMA^11^, with emphasis on the reduction of fugitive dust (street wash-out and control of industrial and construction site sources), mobile fuel type change (diesel to compressed natural gas for buses and trucks), and residential heating source change (coal to liquefied natural gas). However, the efficiency of this act is sometimes questioned by the public, since they still experience severe high pollutant episodes. One intriguing question for the SMA particulate matter (PM; PM_10_, particles with a diameter of 10 µm or less, is used in the study unless specified) concentration is its interannual trend, which recently showed a signal of increase after a long downward trend. Since the early 2000s, annual mean surface PM concentrations have decreased continuously over the SMA and South Korea (Fig. 1). The annual mean concentration was 71.9 μg/m^3^ in 2002, and continued to decrease until it reached its minimum (46.9 μg/m^3^) in 2012, but since then it has begun to rise (51.2 μg/m^3^, 51.9 μg/m^3^, 51.0 μg/m^3^ in 2013, 2014, and 2015, respectively). Although several reasons (e.g., regional transport changes in China^12, 13^ or increased use of diesel-engine passenger cars)^14^ have been suggested for the recent changes, the potential of meteorological variance has not been actively discussed until now.

In addition to the changes in the amount of released emissions of pollutants and their precursors, meteorological conditions are also primary contributors to regional haze and air pollutions^15, 16^. Wind, in its velocity and transport pattern, is one of the major meteorological components that affects surface air pollution^17, 18^. Winds can control vertical mixing and regional ventilation^19^. Persistent stagnant conditions (i.e. calm wind) provide critical conditions that lead to the development of local pollution episodes. Wind direction controls the source-receptor relationship^20^, and it also directly initiates local emissions such as dust storms or sea salt emissions^21, 22^. Large scale transport, both regional or inter-continental, can affect the atmospheric composition by altering their lifetime (e.g. longer lifetime at higher altitudes), and influences global meteorology and climate^23, 24^.

**Figure 2:**
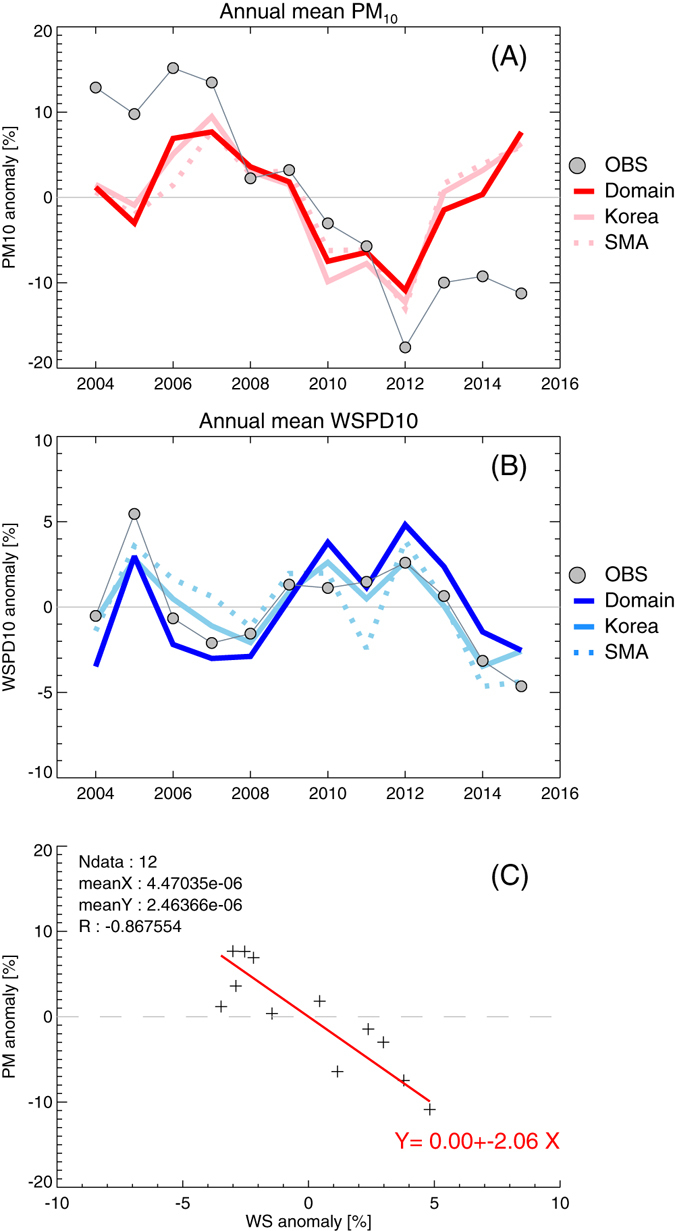
Normalized anomalies of annual mean surface PM concentration (**A**), and annual mean 10-m wind speed (**B**). Red (blue), pink (light blue), and dashed pink (light blue) lines indicate anomalies of modeled PM concentrations averaged over the 9-km domain-wide, Korea (land pixels), and the SMA regions for PM concentrations (for wind speed). Circles indicate observations of surface PM concentrations (247 sites over South Korea) and wind speed (79 sites over South Korea). The scatter plot (**C**) shows a least square regression fit between normalized anomalies of surface PM concentrations and wind speed from the model (9-km domain average).

Identifying the major drivers in the long-term trend of surface PM concentration can be limited due to the intricacy of chemical and meteorological processes. A 12-year simulation with a constant anthropogenic emission inventory was designed to isolate the interannual variation of surface PM concentrations solely due to variations of meteorology (See method). After testing multiple meteorological variables, we found a significant association of wind speed and surface PM concentrations. Normalized anomalies (annual averages subtracted and then divided by the 2004–2015 average) of surface PM concentration and 10-m wind speed averaged over three geographical coverages (i.e. 9-km domain-wide, Korea, and the SMA) are compared (Fig. 2). Noticeably, interannual variations of modeled surface PM concentrations show very similar patterns in all regions. The observed PM, averaged over 247 sites in South Korea, shows similar but slightly different interannual variability. Its year-to-year variation pattern is very similar to that of modeled variation; a small drop in 2004, strong positives in 2005–2007, strong negative in 2012, and an increase in 2013–2014. Its general pattern, however, is mostly positive in early years and negative in later years. It thus seems reasonable to say that the observations show mixed signals; likely a combination of a short-term year-to-year fluctuations (caused by meteorological changes) and a long-term decline (caused by changes in anthropogenic emissions)^25^.

Normalized anomalies of 10-m wind speed, on the other hand, show good agreement between observations (averaged over 79 sites in South Korea) and modeled wind speed over three regions, demonstrating that the model reasonably simulated interannual variations of the wind field. The key feature from the interannual variations of surface PM concentrations and surface wind is that they show totally opposite phases in their interannual variabilities. It should be noted that the interannual variations of PM concentrations, in all three regions, are best explained by the changes of domain-wide averaged wind speed compared with smaller scale averages. This might imply that the controlling mechanism is not on a local scale (e.g. sea breeze or local circulation) but rather synoptic scale activities (e.g. frontal passage). Considering the efficiency of the wipe-out mechanism by cold frontal activities, the annual wind speed variability seems to represent the efficiency of regional ventilation^26^. Northeastern Asia is a routine passage of mid-latitude cyclogenesis, initiated on the lee side of the Altai-Sayan Mountains^27^. The role of Altai-Sayan cyclogenesis, as an efficient cleaning mechanism of pollutants over northern China and Korea during cool season, is demonstrated and discussed in Kim *et al*.^28^. The change of summertime circulation (i.e. East Asia summer monsoon) on regional pollution is also discussed in Zhu *et al*.^29^.

Scatter plots of normalized anomalies between wind speed and surface PM concentration from the model (Fig. 2C) clarify their correlation. A least square regression line fit is PM [%] = −2.1 WS [%], with strong Pearson correlation coefficient, R = −0.86. During the 2004–2015 period, the PM concentration anomaly varied between −10.9% and 7.7%, while the wind speed anomaly varied between −3.5% and 4.8%. The range of variability in PM concentration is impressive since it is almost compatible to total long-term changes; that is ~−2% per year or ~−24% during 2004–2015. Remarkably, even with excellent agreement between modeled and observed interannual anomalies in surface PM concentration and wind speed, a direct comparison between observed PM concentration and observed wind speed variability shows poor correlation (i.e. R = 0.07). It clearly shows the advantage of the emission-isolating modeling approach, otherwise the meteorology-driven interannual variability of PM concentration seems to be overshadowed by emission reduction in the long-term trends.

**Figure 3:**
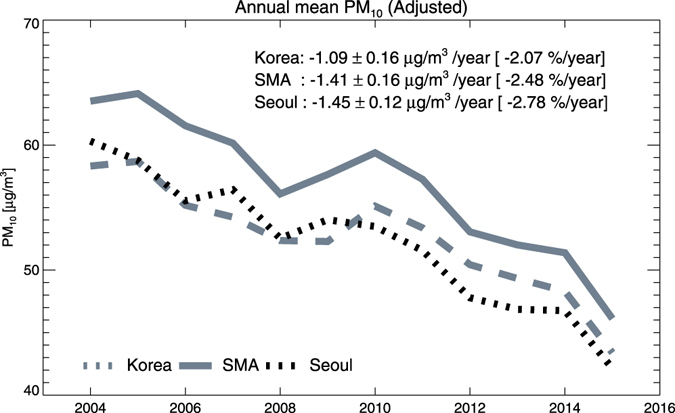
Time series of adjusted surface PM concentrations over Seoul, the SMA, and South Korea. Modeled fractional interannual anomalies (Fig. 2) are applied to remove meteorology-driven interannual variances from the original PM concentrations in Fig. 1.

Finally, we tried to remove the meteorology-driven variances from the original PM concentrations. Fractional interannual anomalies from Fig. 2A are multiplied to an overall mean of PM concentrations from each region, and then subtracted from the original annual mean concentrations. Considering the uncertainties from the original model bias, using the relative variances instead of absolute concentrations from the model is a more robust approach^30^. Adjusted annual PM concentrations, with meteorology-driven variances removed, are shown in Fig. 3 in relation to Seoul, the SMA, and South Korea. The most surprising change of the annual PM concentrations, compared with those of Fig. 1, is their trend in recent years. The increase since 2012, which had frustrated many emission policy-makers in the South Korean government, seems to have disappeared. With adjusted concentrations, we estimate that the annual surface PM concentrations have decreased by −1.45 ± 0.12 μg/m^3^, −1.41 ± 0.16 μg/m^3^, and −1.09 ± 0.16 μg/m^3^ per year, for Seoul, the SMA, and South Korea, respectively. Apparently, the decreasing rate in Seoul is faster than the national average, implying the effectiveness of special emission control efforts in the locale, including replacing diesel buses and diesel garbage trucks with natural gas vehicles^31, 32^.

The local minimum of 2008 in the adjusted PM concentrations is noteworthy. The most likely explanation for this decrease is the impact of the global recession during 2008–2009, as indicated by several previous researches^33, 34^. At this point, we are not able to distinguish if the impact is due to changes caused by the South Korean economy’s experience of the global recession, or due to changes of transported pollutants and precursors by Chinese economic changes. The case of 2015 is also interesting. PM concentration was higher in early 2015 (64.1 μg/m^3^ in 2014 Jan-Mar and 72.5 μg/m^3^ in 2015 Jan-Mar), but the annual mean is lower than that of 2014 because its Spring-to-Fall concentration is very low, even with the weakest wind speed observed. This is likely an extraordinary case by the impact of the Middle East Respiratory Syndrome (MERS), which has seriously impacted the South Korean economy and society^35^. Although further investigations are necessary, these cases in 2008 and 2015 may provide interesting examples of the socio-economic impact on the environment, in addition to emission control policy-driven impact. Three factors, long-term trend by emission control, short-term variation by meteorology, and sporadic offsets by unexpected social or economic episodes, seem to be affecting PM concentrations in South Korea.

To conclude, we found that there is a strong correlation between variations of surface PM concentrations in South Korea and variations of wind speed. This study addresses several important implications. First, interannual variation of meteorological conditions has the strong potential to affect long-term trends in surface PM pollution events. At this point, we do not have any evidence that the reduced wind speed is part of long-term change or just short-term fluctuation in general circulations or ventilation patterns. However, it is worth further investigation since the frequency of mid-latitude cyclones can be reduced in warmer weather conditions, as suggested through observations^36–39^ and models studies^19, 40–42^. Mickley *et al*.^19^ used the Goddard Institute of Space Studies model to demonstrate that the severity and duration of summertime regional pollution can increase due to a decline in the frequency of mid-latitude cyclones under future warmer climates. This may provide a key feature to understand the frequent occurrence of severe haze episodes not only in South Korea but also in East Asia, especially in northern China. Yang *et al*.^43^ also demonstrated haze days over eastern China has increased between 1980 and 2014, and argued that the weakening of winds is the dominant factor leading the decadal increase.

Second, accurate simulation of surface wind field, especially in terms of wind speed, seems to be critical for an accurate regional air quality model and forecast. Analysis suggests a 5% change in annual wind speed is associated with 10% annual surface PM concentration. In many air forecast simulations in South Korea, including the current long-term simulation used in this study, modeled surface winds tend to be overestimated (2.2 m/s observed and 3.3 m/s modeled) and modeled surface PM concentrations are generally underestimated (52.6 μg/m^3^ observed and 27.9 μg/m^3^ modeled). Underestimation of surface PM concentration is somehow expected, due to the lack of natural emission sources, e.g. Asian dust and wildfire emissions. However, the possible contribution of overestimated surface wind speed should also be considered. If we apply the correlation PM [%] = −2.1 · WS [%] from Fig. 2C, then reducing wind speed from 3.3 m/s to 2.2 m/s (i.e. −30%) can increase PM concentration from 27.9 μg/m^3^ to 45.5 μg/m^3^ (i.e. +63%), which can explain the 71% of current PM simulation bias (i.e. (45.5–27.9)/(52.6–27.9) = 71%).

Third, most importantly, emission control efforts from the South Korean government and community, as well as neighboring countries, seem to be effective. However, changes in meteorological conditions seem to offset those efforts. Recent space-borne observations also confirm a considerable decrease of NO_2_ and SO_2_ vertical column densities over the SMA, South Korea, and northeastern China (i.e. the Beijing-Tianjin-Hebei region)^44, 45^.

## Methods

In order to investigate the interannual variation of surface PM concentration in the SMA, we conducted a 12-year simulation using a regional air quality modeling system. The Weather Research and Forecasting Model (WRF), the Sparse Matrix Operator Kernel Emission (SMOKE), and the Community Multiscale Air Quality (CMAQ) models were utilized to simulate meteorology and chemistry over East Asia (27-km domain) and over South Korea (9-km domain). The 27-km East Asia domain was chosen to cover possible interactions among international emissions from neighboring countries (e.g. China, Japan, and North Korea), and provides boundary conditions for the 9-km simulation. The 9-km domain then covers the entire of South Korea. The geographical coverage of domains and locations of surface monitoring sites are shown in the supplementary material.

For meteorology, WRF (version 3.3.1)^46^ was initiated with the National Center for Environmental Protection (NCEP) Final Analysis (FNL) product^47^. Shuttle Radar Topography Mission (SRTM) Digital Elevation Model (DEM) with 90-m resolution and Korean Ministry of Environment Land Use Land Cover data were used for terrain and surface land type, respectively. The Meteorology-Chemistry Interface Processor (MCIP, version 3.6) was used as a preprocessor for CMAQ simulation. CMAQ (version 4.7.1)^48^ with AERO5 aerosol module^49^ and the Statewide Air Pollution Research Center version 99 (SAPRC99)^50^, were chosen as the gas-phase chemical mechanism.

An emission inventory set, Clean Air Policy Support System (CAPSS) 2007 for South Korea and Intercontinental Chemical Transport Experiment Phase-B (INTEX-B) 2006 emission inventory for other Asian countries, were used for the 12-year simulation to isolate the impact of interannual variability of anthropogenic emissions. At the same time, an additional choice for the recent emission inventory set, the Model Inter-Comparison Study for Asia (MICS-Asia) 2010 and CAPSS 2010, was also used to check whether there was any dependency on the choice of emission inventory. However, the simulated surface PM concentrations from both emission inventory sets show almost identical interannual fluctuations of PM concentrations, as expected.

The 2006 INTEX-B emission inventory provides emissions for eight species, SO_2_, NO_x_, CO, NMVOC, PM_10_, PM_2.5_, BC, and OC. Data are available for four emission sectors (power plants, industry, residential, and transportation) over 22 countries and regions in Asia (http://mic.greenresource.cn/intex-b2006)^51^. For domestic South Korean emissions, the CAPSS emission inventories were utilized, which provided emission information on point for on-road and non-road emission sectors. Emission sources are classified into four levels: 12 upper level categories, 54 intermediate level categories, 312 lower level categories, and 527 detail level categories. The upper level categories include combustion in energy industries (point sector), non-industrial combustion plants, combustion in manufacturing industries, production processes, storage and distribution of fuels (point and area sector), solvent use, other mobile sources and machinery (mobile sector), waste treatment and disposal (point sector), agriculture, other sources and sinks (area sector), and fugitive dust (mobile and area sector) categories. Emissions for CO, NOx, SOx, PM_10_, and VOCs are available for each upper level category except fugitive dust emissions^5^. The Model of Emissions of Gases and Aerosols from Nature (MEGAN version 2.04)^52^ was used to prepare biogenic emissions. MCIP-processed ground reaching solar radiation and 2-m temperature were used to adjust hourly biogenic emissions such as isoprene and monoterpenes. Other vegetation data like leaf area index, plant function type, and emission factors were also used as released with MEGAN v2.04. Biomass burning and dust emissions were not included.

The modeling configuration was tested for multiple years as part of the Integrated Multi-Scale Air Quality Study for Korea (IMAQS-K) system, which was initially developed as a prototype of the official national air quality forecast system in South Korea. The IMAQS-K system has been operational since 2012. Descriptions on physical options are provided in Kim *et al*.^53^, and basic model performance evaluations for 12-year period are provided in the supplementary materials. Model performed well in reproducing spatial and temporal variations of meteorology and chemical components. While total particulate matter concentration generally underestimates in the model, current analysis, using percent change of inter-annual variation, is designed to be less affected by the model bias.

### Surface monitoring data

Hourly observations of surface PM concentrations and wind speed were obtained from NIER and KMA, respectively. PM measurements, based on the beta-ray absorption method^54^, were collected from 247 urban air monitoring network sites, and wind data were collected from 79 meteorological monitoring sites. Locations of monitors are shown in the supplementary material.

### Note on other controlling factors

(1) A recent study on the regional emission attribution to the South Korea suggests that the relative attribution of foreign emission sources is not sensitive to inter-annual variation of meteorology^55^, contributing around 60% of the SMA PM concentration. Combined with the current declining trend of anthropogenic emissions from China, we can rule out the possibility of the increased efficiency of Chinese emissions transport. (2) The number of diesel vehicles in South Korea has increased 13% from 2012 to 2014. Since non-truck diesel vehicles emit 5.6% of total NO_x_ emission in South Korea^5^, resultant impact is small (<1% of total PM). However, careful and continuous monitoring of diesel vehicle emissions is necessary due to their high uncertainty in emissions factors as we have learned from the Volkswagen emission scandal^56^.

## Electronic supplementary material

Supporting Information
