# Supplementary material for: Recent increase of surface particulate matter concentrations in the Seoul Metropolitan Area, Korea
Source: Sci Rep. 2017 Jul 5;7:4710. doi: 10.1038/s41598-017-05092-8 (PMC5498658; doi:10.1038/s41598-017-05092-8)

**Supporting Information**

**Recent increase of surface particulate matter concentrations in the Seoul Metropolitan Area, Korea**

Hyun Cheol Kim^1,2^, Soontae Kim^3*^, Byeong-Uk Kim^4^, Chun-Sil Jin^5^, Songyou Hong^6^, Rokjin Park^7^, Seok-Woo Son^7^, Changhan Bae^3^, MinAh Bae^3^, Chang-Keun Song^8^, and Ariel Stein^1^


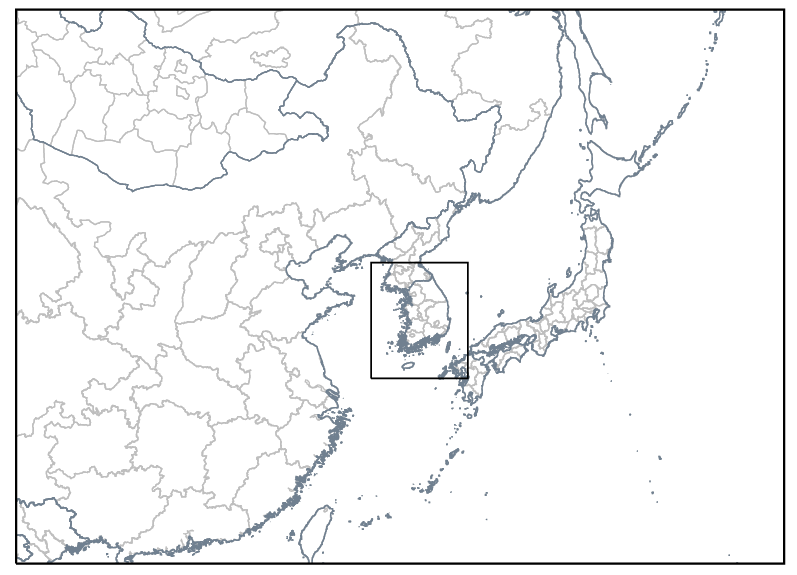


Figure S1 Spatial coverage of 27-km East Asia domain. Map is generated using Interactive Data Language version 8.2.1 (Harris Geospatial Solutions, [http://harrisgeospatial.com](http://harrisgeospatial.com/)) with Global Administrative Areas ([http://gadm.org](http://gadm.org/)) map data.


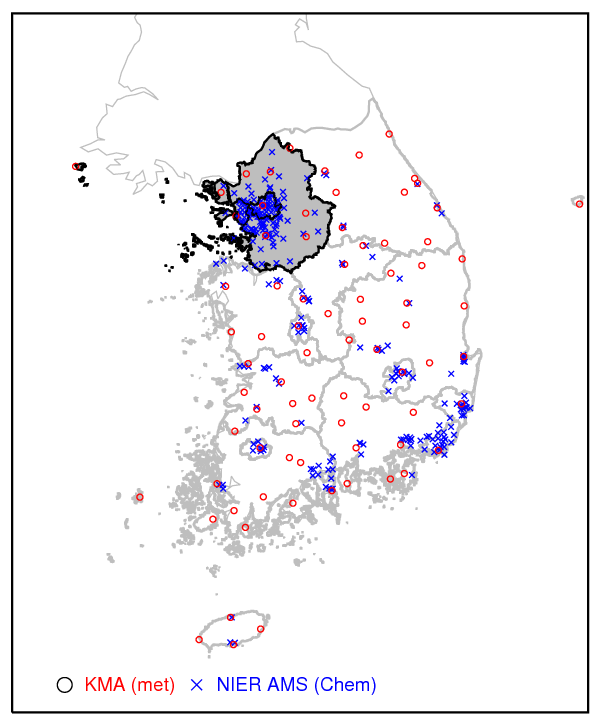


Figure S2 spatial coverage of 9-km South Korea domain. Circles indicates KMA surface monitoring sites (meteorology, 79 sites), and X marks indicate NIER AMS sites (chemistry, 247 sites). Shaded area indicates the Seoul Metropolitan Area. Map is generated using Interactive Data Language version 8.2.1 (Harris Geospatial Solutions, [http://harrisgeospatial.com](http://harrisgeospatial.com/)) with Global Administrative Areas ([http://gadm.org](http://gadm.org/)) map data.


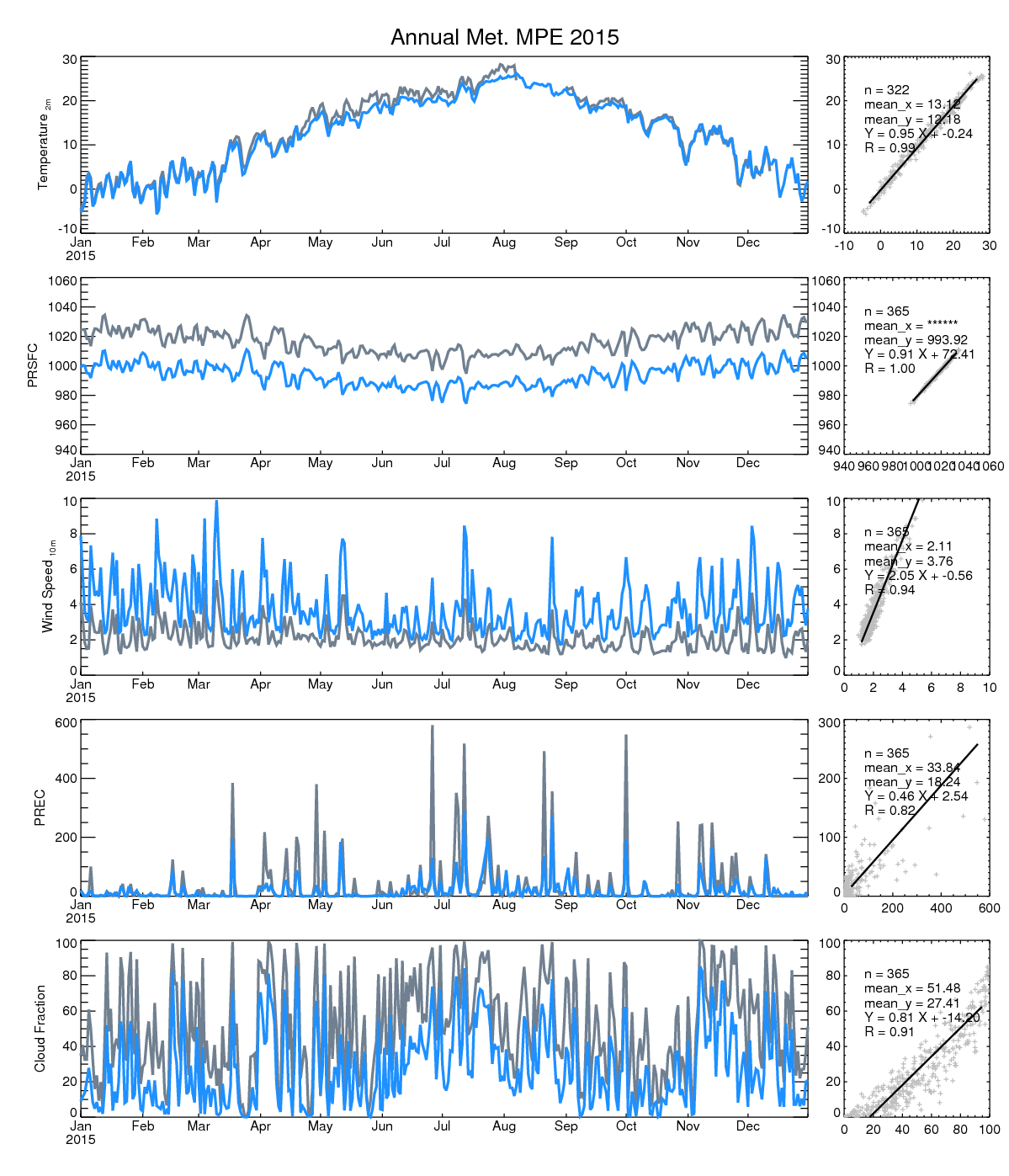


Figure S3 Annual model performance evaluations for meteorological variables for 2015. 2-m temperature, surface pressure, 10-m wind speed, precipitation and cloud fractions are shown. Observations are collected from 79 Korean Meteorological Administration surface monitoring sites. All evaluations during 2004-2015 are available at http://imaqs.ajou.ac.kr/hkim/pub/SREP-16-49878-T/.


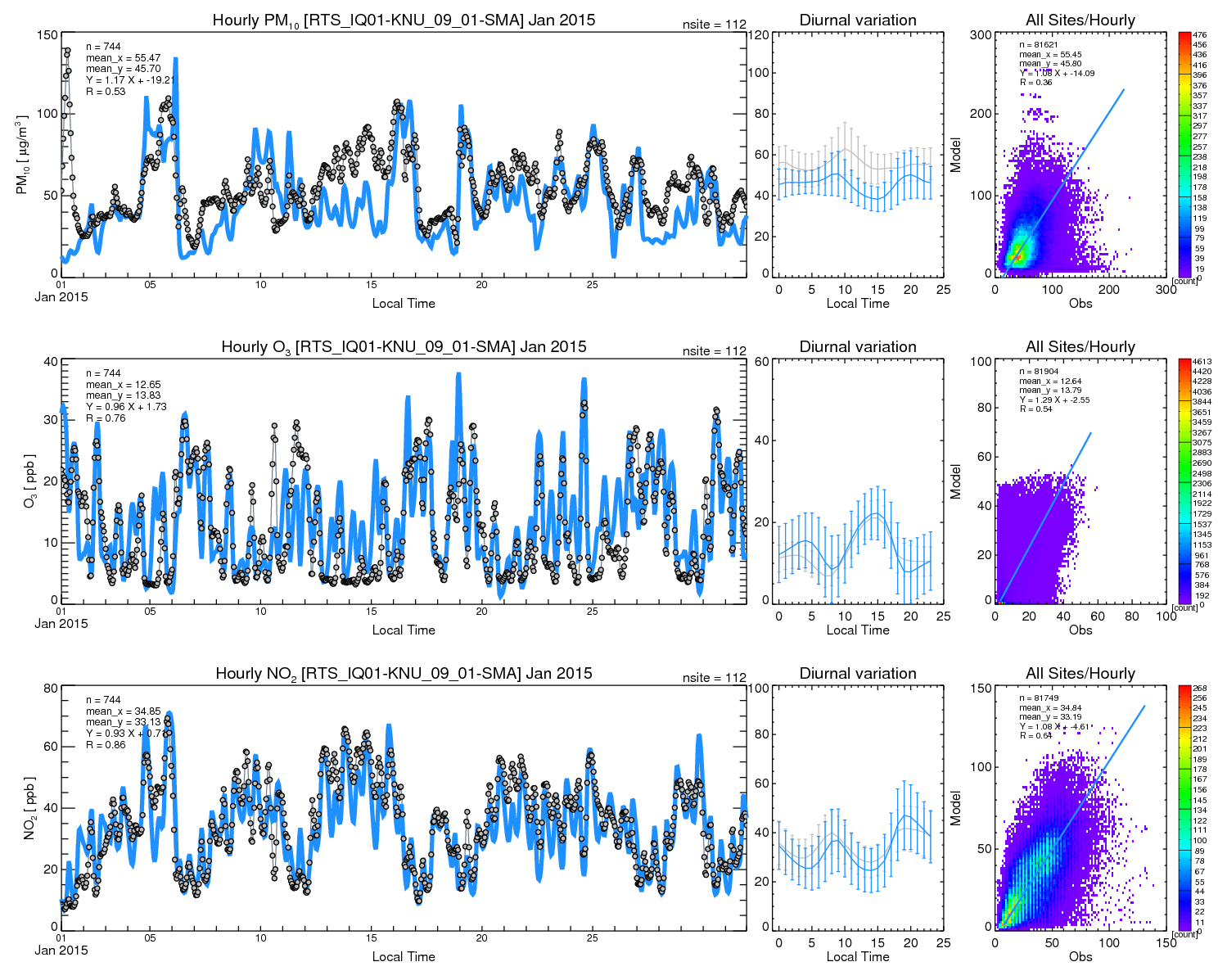


Figure S4 Monthly model performance evaluations for surface PM_10_, O_3_ and NO_2_ during January - December 2015. Observations are collected from National Institute of Environmental Research surface monitoring sites in the Seoul Metropolitan Area, Korea. All monthly evaluations during January 2004 ~ December 2015 are available at http://imaqs.ajou.ac.kr/hkim/pub/SREP-16-49878-T/.


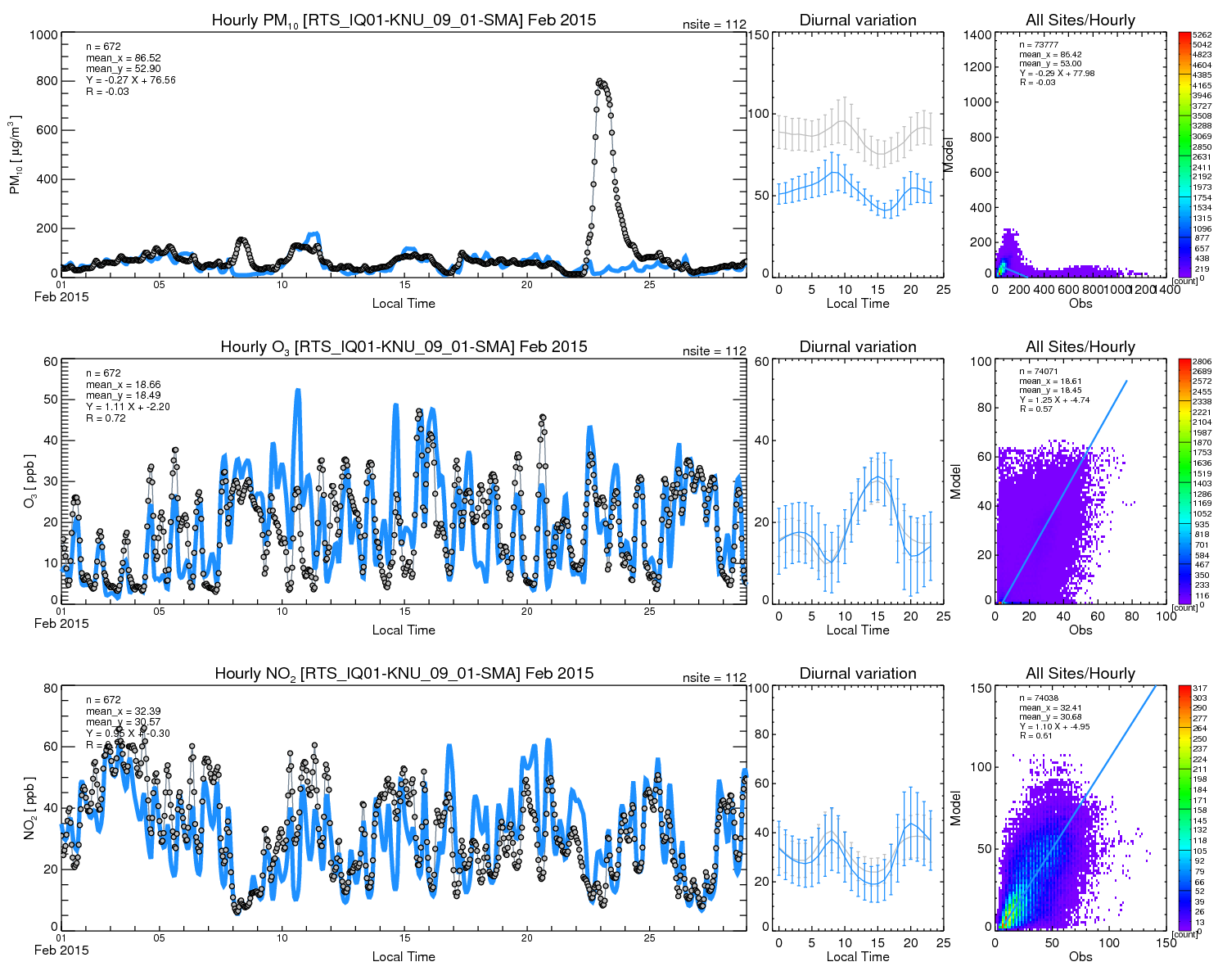


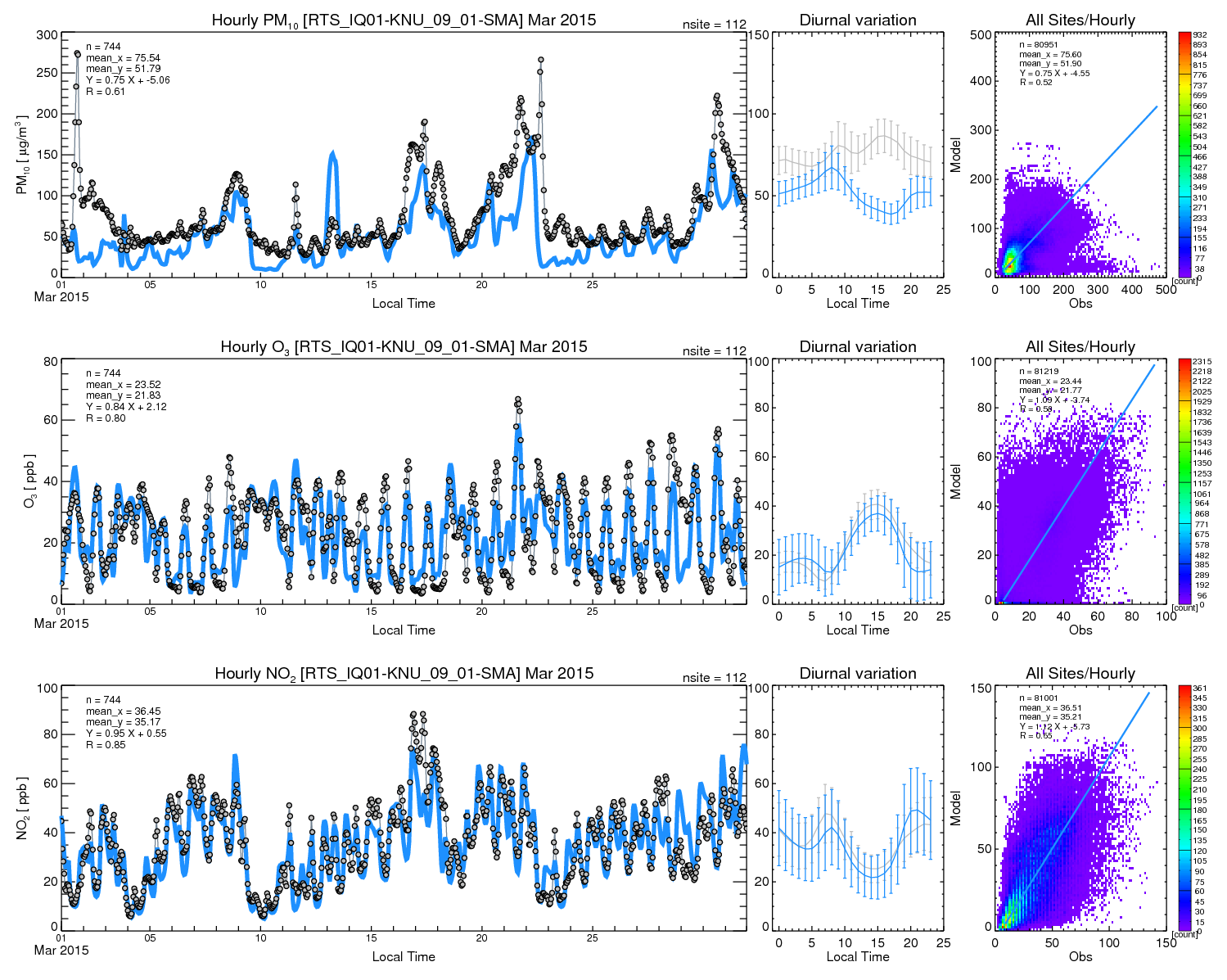


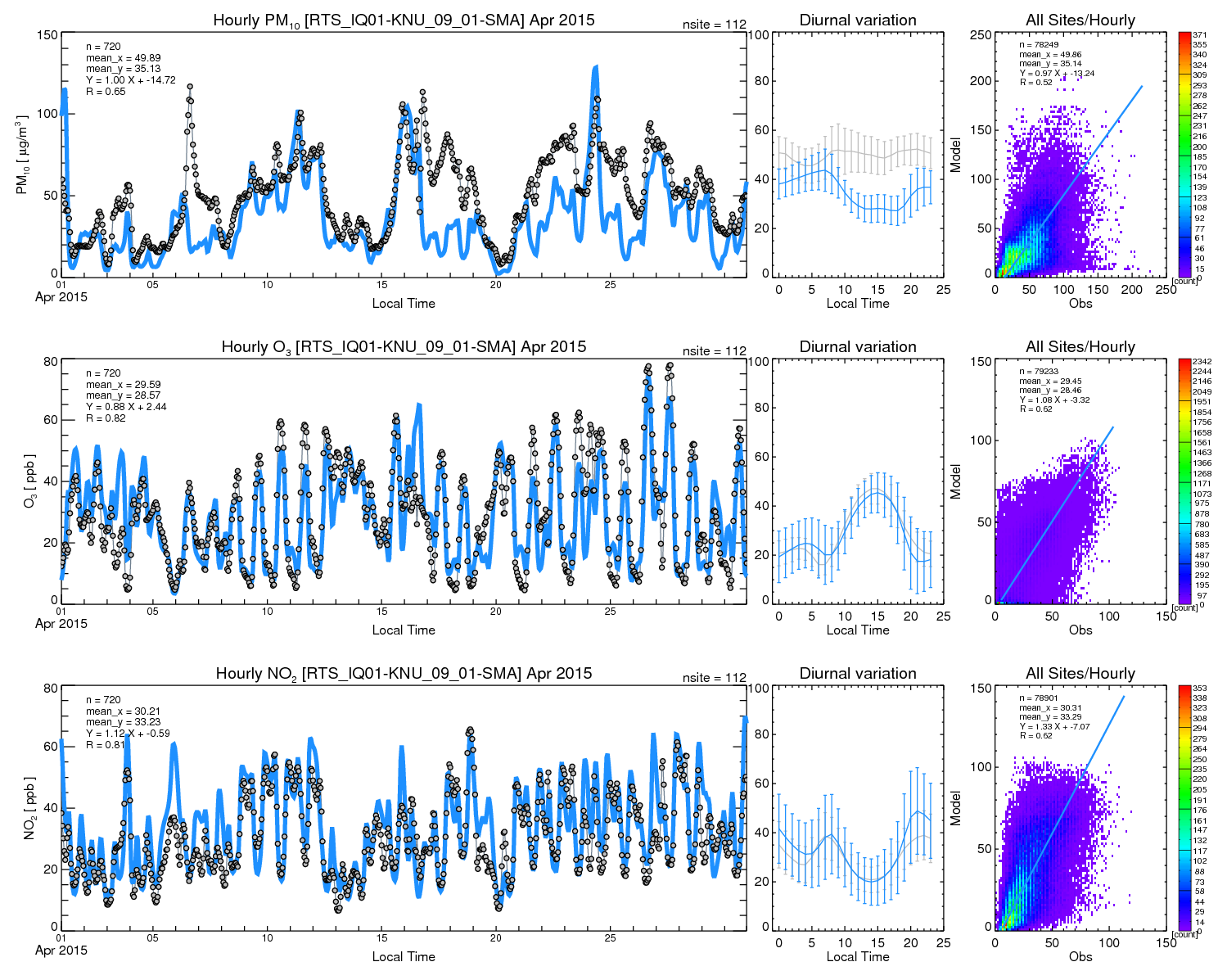


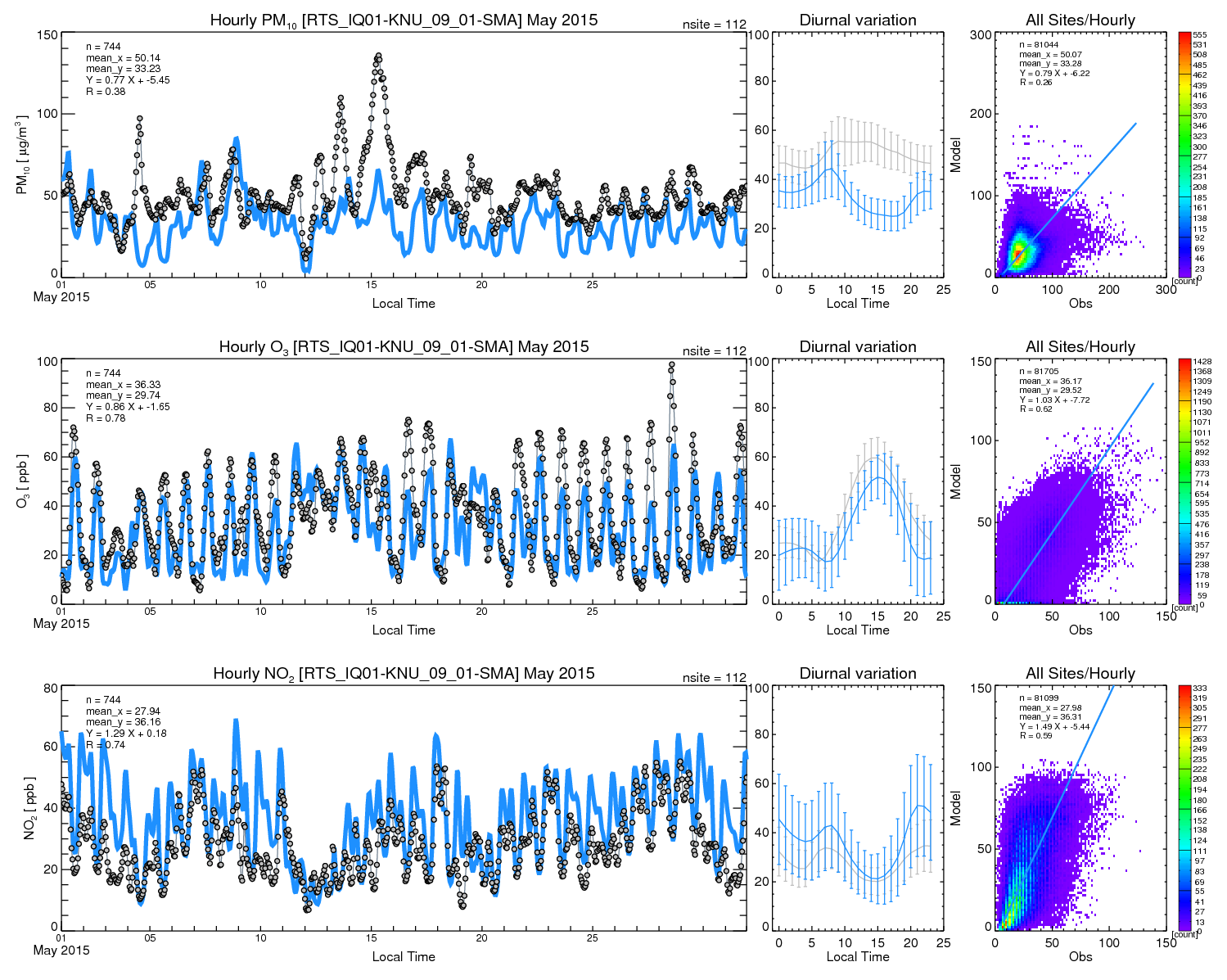


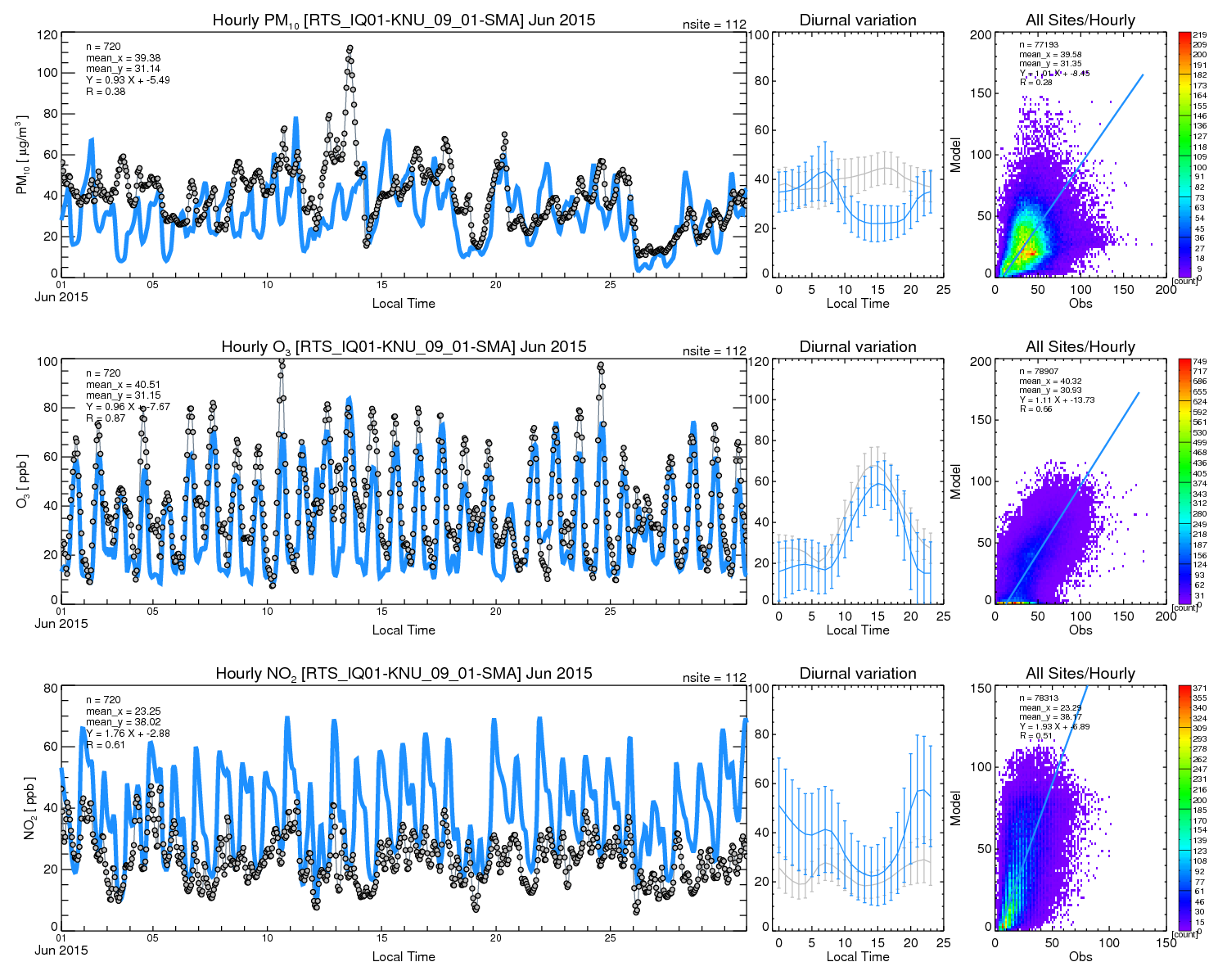


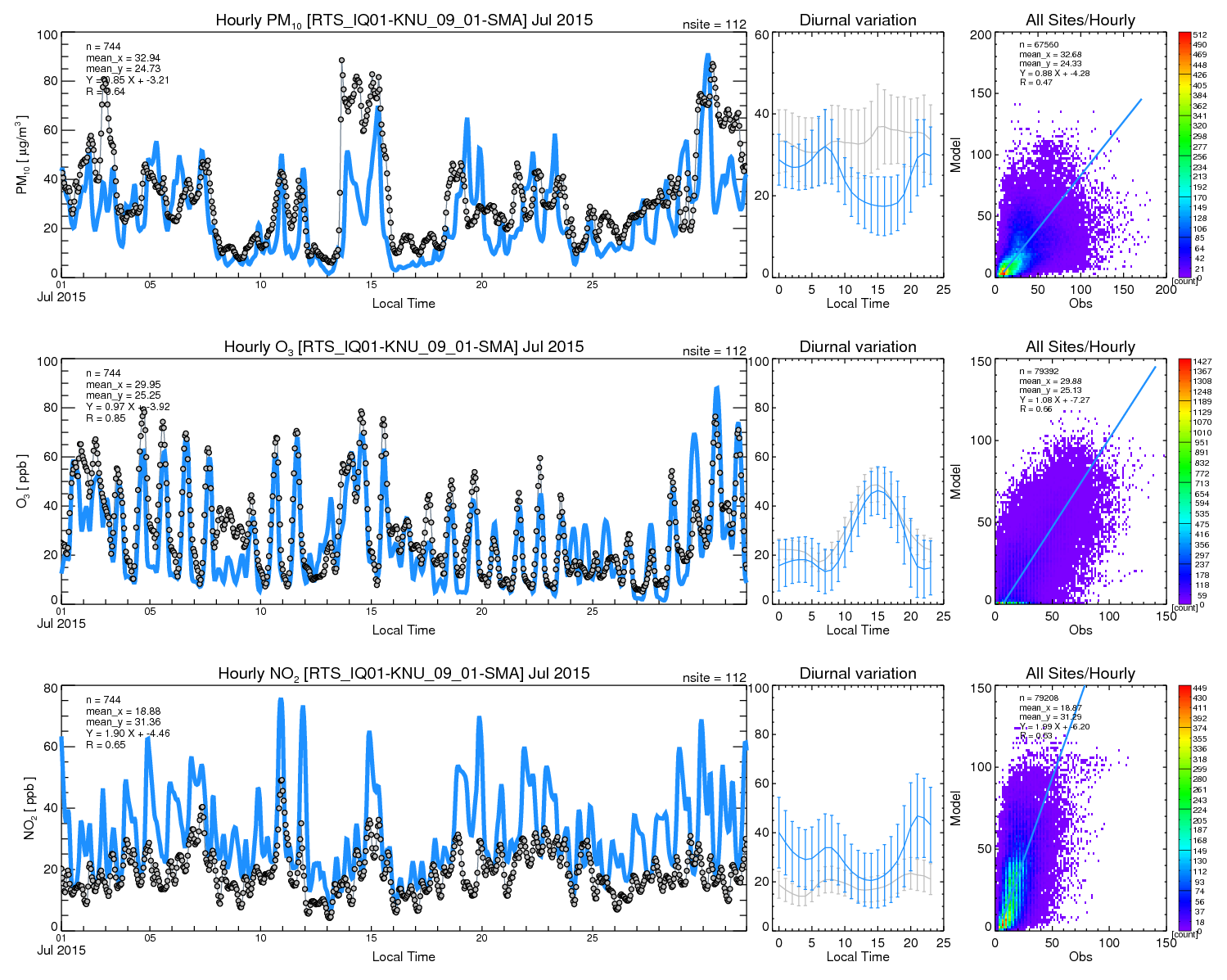


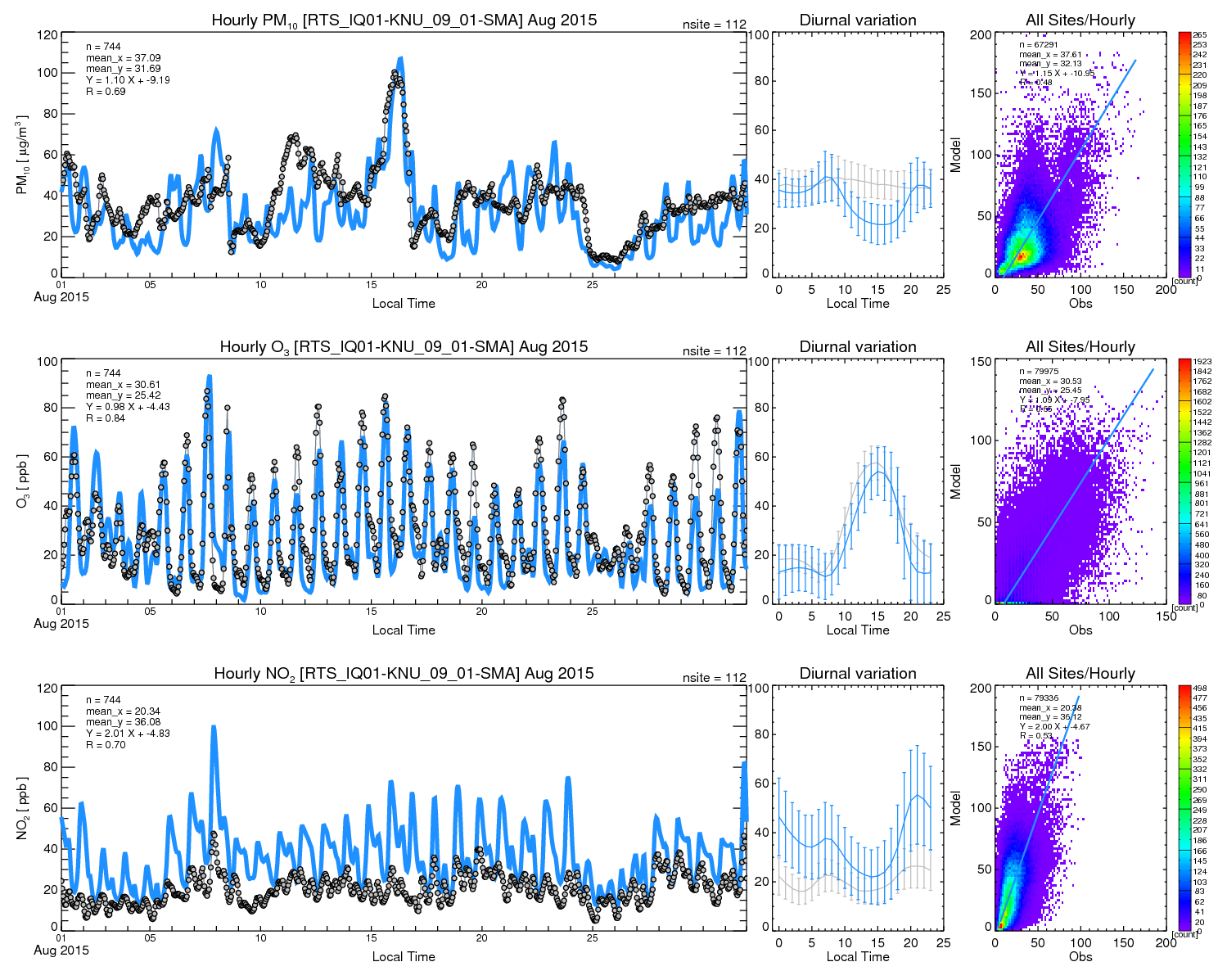


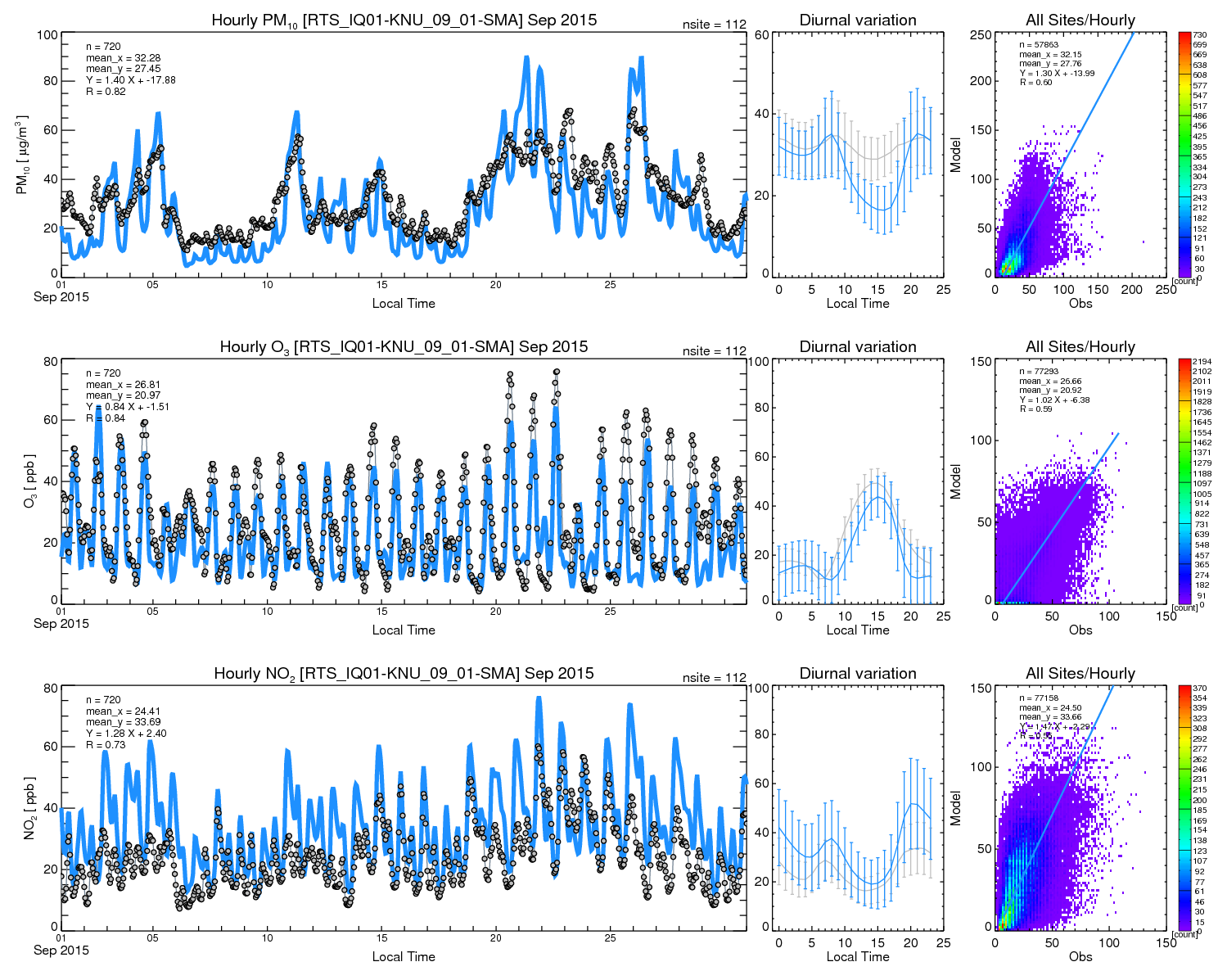


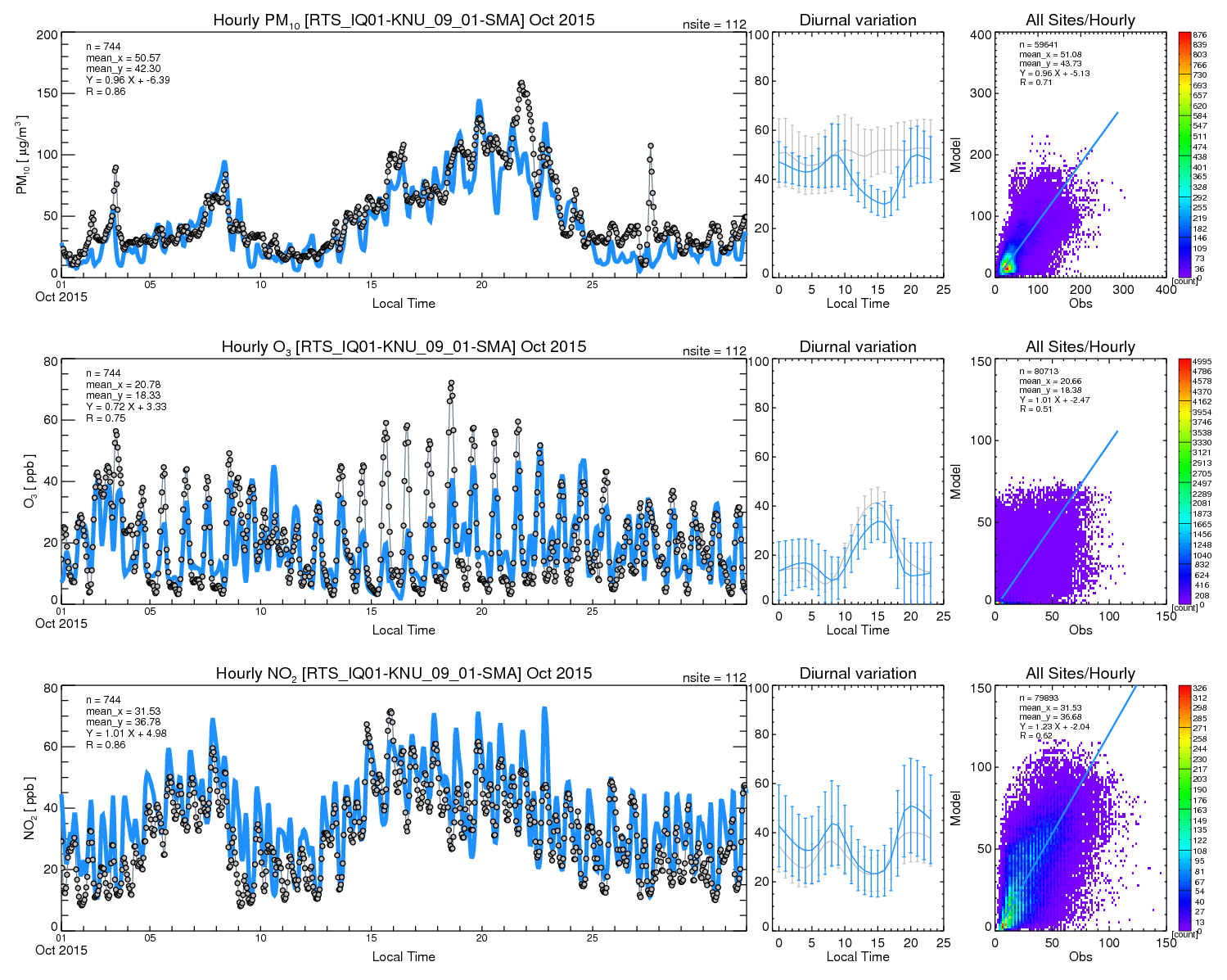


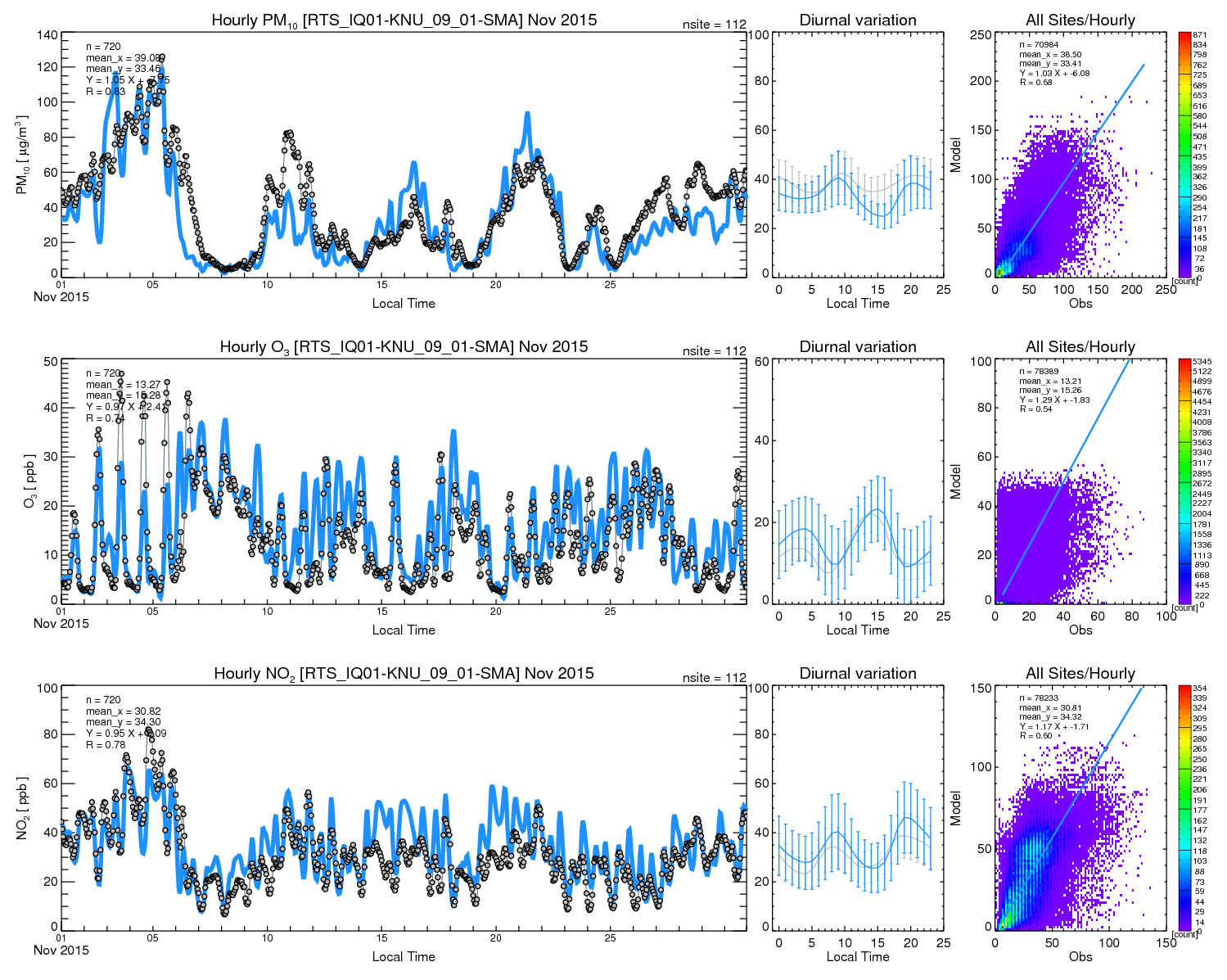


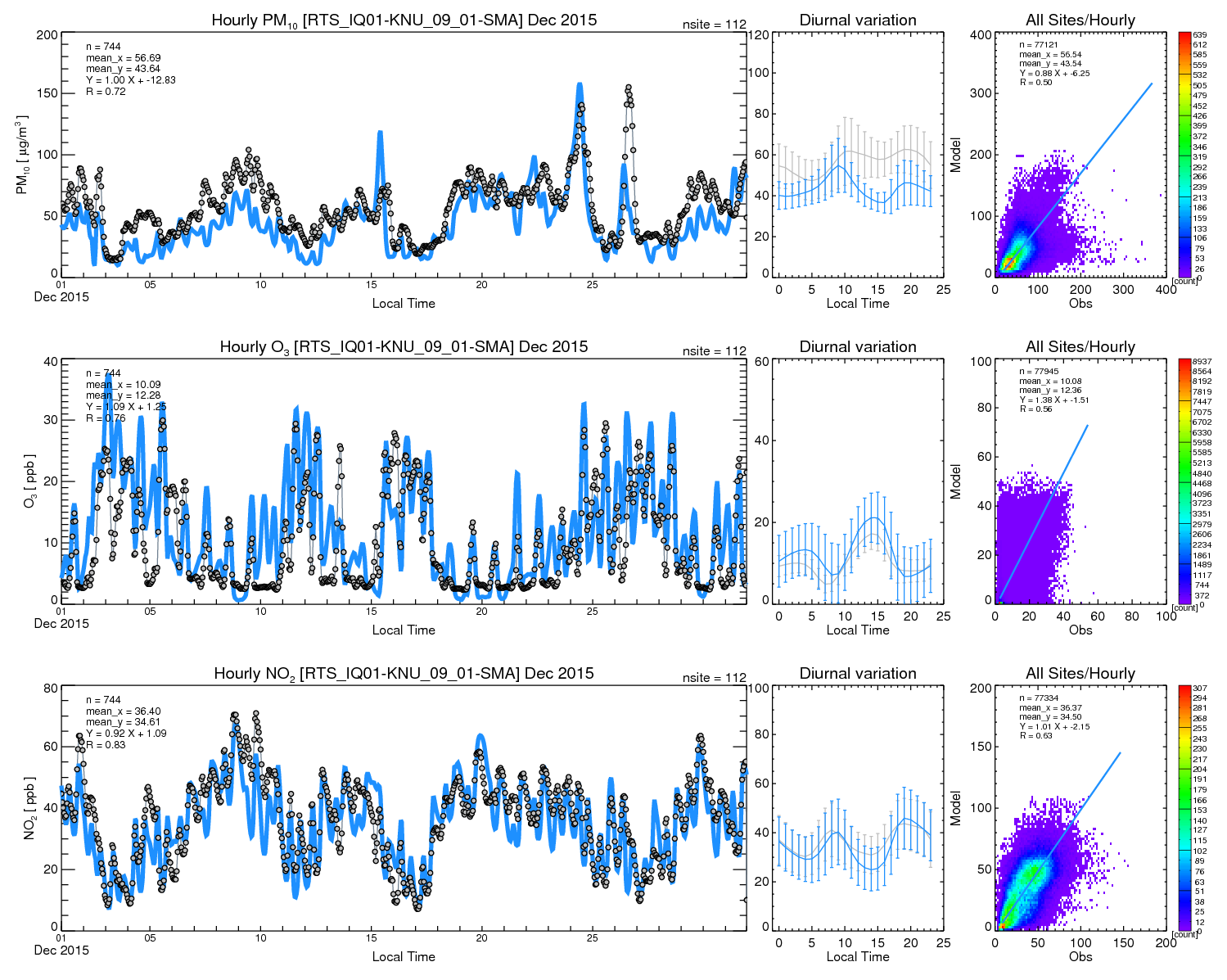

Supplement: Supplementary file 1 — Supporting Information [file 41598_2017_5092_MOESM1_ESM.doc]
